# Characterization of GLPG0492, a selective androgen receptor modulator, in a mouse model of hindlimb immobilization

**DOI:** 10.1186/1471-2474-15-291

**Published:** 2014-09-03

**Authors:** Roland Blanqué, Liên Lepescheux, Marielle Auberval, Dominique Minet, Didier Merciris, Céline Cottereaux, Philippe Clément-Lacroix, Philippe Delerive, Florence Namour

**Affiliations:** GALAPAGOS SASU, 102 Avenue Gaston Roussel, 93230 Romainville, France

**Keywords:** Selective androgen receptor modulator, Immobilization, Muscle wasting, Anabolism

## Abstract

**Background:**

Muscle wasting is a hallmark of many chronic conditions but also of aging and results in a progressive functional decline leading ultimately to disability. Androgens, such as testosterone were proposed as therapy to counteract muscle atrophy. However, this treatment is associated with potential cardiovascular and prostate cancer risks and therefore not acceptable for long-term treatment. Selective Androgen receptor modulators (SARM) are androgen receptor ligands that induce muscle anabolism while having reduced effects in reproductive tissues. Therefore, they represent an alternative to testosterone therapy. Our objective was to demonstrate the activity of SARM molecule (GLPG0492) on a immobilization muscle atrophy mouse model as compared to testosterone propionate (TP) and to identify putative biomarkers in the plasma compartment that might be related to muscle function and potentially translated into the clinical space.

**Methods:**

GLPG0492, a non-steroidal SARM, was evaluated and compared to TP in a mouse model of hindlimb immobilization.

**Results:**

GLPG0492 treatment partially prevents immobilization-induced muscle atrophy with a trend to promote muscle fiber hypertrophy in a dose-dependent manner. Interestingly, GLPG0492 was found as efficacious as TP at reducing muscle loss while sparing reproductive tissues. Furthermore, gene expression studies performed on tibialis samples revealed that both GLPG0492 and TP were slowing down muscle loss by negatively interfering with major signaling pathways controlling muscle mass homeostasis. Finally, metabolomic profiling experiments using ^1^H-NMR led to the identification of a plasma GLPG0492 signature linked to the modulation of cellular bioenergetic processes.

**Conclusions:**

Taken together, these results unveil the potential of GLPG0492, a non-steroidal SARM, as treatment for, at least, musculo-skeletal atrophy consecutive to coma, paralysis, or limb immobilization.

**Electronic supplementary material:**

The online version of this article (doi:10.1186/1471-2474-15-291) contains supplementary material, which is available to authorized users.

## Background

Maintenance of skeletal muscle mass is mainly achieved by a homeostatic balance between muscle regeneration, protein synthesis and protein degradation. This tight balance is significantly altered during aging, leading to muscle wasting coupled to a decline in function over time named sarcopenia[1]. In addition, skeletal muscle atrophy occurs in many chronic conditions such as cancer cachexia, sepsis, diabetes, renal failure and arthritis[2, 3]. Mechanistic studies performed in rodent models of muscle atrophy as well as in patients revealed that atrophy is an active process regulated by specific signaling pathways and transcriptional programs (See[4] for review). Clusters of genes similarly up or down-regulated in the atrophied muscle under various conditions are referred as atrogenes[5]. The rapid induction of the muscle-specific ubiquitin ligases, MAFbx/atrogin-1 and muscle ring finger 1 (MurF1) gene expression in response to atrophic stimuli revealed the activation of protein catabolism via the Ubiquitin (Ub)26S proteasomal pathway as an early event leading to muscle atrophy[6–8]. Furthermore, mice deficient for Atrogin-1 or MurF1 were shown to be resistant to some extent to denervation induced atrophy[6]. Finally, Atrogin-1 and MurF1 were reported to be involved in the degradation of both regulatory and structural proteins including MyoD, Calcineurin A and myosin, respectively[9–12]. In addition to the Ubiquitin-proteasome pathway, autophagy has also been shown as a major signal activated at the transcriptional level and leads to protein catabolism (See[4] for review). The lysosome proteases, Cathepsin-L and LC3-II were reported to be increased in rodent models of muscle atrophy[13, 14]. FoxOs have been identified as key transcription factors at the crossroads of both protein breakdown via the regulation of Atrogin-1 and MurF1 gene transcription[8, 15] and protein synthesis via the regulation of 4EBP1 and the subsequent inhibition of the mTOR signaling pathway[16]. Disruption of the subtle balance between protein synthesis and degradation during muscle atrophy involves the suppression of bioenergetic pathways associated to mitochondrial functions and controlled by PGC-1α whose expression is sharply reduced in various models of muscle wasting[17–19].

Despite our growing understanding of the molecular mechanisms controlling muscle atrophy, there is still an unmet need for therapies that prevent or reverse muscle atrophy resulting into a significant improvement of physical function in patients. A large number of studies have established the beneficial impact of androgen treatment on both muscle mass and strength[20, 21]. Androgens control a broad spectrum of physiological processes mainly via the nuclear androgen receptor (AR), a member of the nuclear receptor super-family[22]. However, treatment with androgens such as testosterone is associated with potential cardiovascular and prostate cancer risks[23]. Furthermore, alteration of circulating levels of androgens or modulation in receptor function lead to multiple disorders such as hypogonadism, muscle wasting, cachexia, osteoporosis, loss of reproductive functions and prostate cancers[23]. Selective androgen receptor modulator (SARM), synthetic compounds designed to demonstrate tissue-specific action for muscle and bone represent an alternative to testosterone therapy (See[24, 25] for review). We recently identified GLPG0492 as a non-steroidal SARM[26]. This molecule displays an excellent selectivity profile (>500-fold in binding assays) versus members of the steroid receptor family and behaves as a partial AR agonist in cell-based assays[26]. GLPG0492 has been previously tested in a standard castrated male rodent model in which it demonstrated after oral dosing robust anabolic activity on levator ani (LA) muscle comparable to testosterone propionate, but dissociated from the androgenic activity on ventral prostate. The dose displaying 50% activity on LA was 0.75 mg/kg/day, while at the maximum 30% activity can be achieved on prostate at the highest dose tested[27]. So, GLPG0492 has demonstrated a robust selectivity for muscle versus prostate, as SARM[26, 27].

To determine the therapeutic potential of GLPG0492 for muscle wasting-associated diseases, we evaluated this compound in a mouse model of hindlimb immobilization and compared its effects to testosterone propionate as reference. In this model, GLPG0492 attenuated the loss of muscle mass induced by immobilization by reducing, at least in part, fiber atrophy. Furthermore, gene expression studies revealed that GLPG0492 negatively antagonized transcriptional programs induced by immobilization. Finally we look for a metabolomic signature which might be considered as a bona fide biomarker of target engagement in future clinical studies. Taken together, these results revealed the potential of GLPG0492 as treatment for disuse (e.g. coma, paralysis, hospitalization, limb immobilization).

## Methods

### Reagents

GLPG0492 was synthesized by the medicinal chemistry department at Galapagos and was determined to be >99% pure by HPLC and/or NMR analysis[26]. Testosterone proprionate was purchased from Sigma (France). Dosing solutions of GLPG0492 and TP were prepared as solutions in a vehicle of 5% Ethanol and 95% Corn oil.

### Animal studies

Experimental protocols were approved by the Galapagos Institutional Animal Care and Use Committee. Male BALB/cj mice (10 weeks old; n = 10/group, Janvier Labs) maintained on a standard chow diet, were subjected to unilateral hindlimb plaster casting (day 0) as previously described[28]. Intact or immobilized mice were treated for 7 days with increasing doses of GLPG0492 (0.3, 3 and 10 mg/kg/d) or testosterone-propionate (TP: 1 mg/kg/d) or vehicle (5% Ethanol/95% Corn oil) by subcutaneous administration. At the end of the treatment period (5 h after the last dose), mice were sacrificed by decapitation and blood was recovered for serum preparation. Gastrocnemius and tibialis from both immobilized and contralateral legs were collected, weighed, and snap-frozen in liquid nitrogen.

### Bio-analysis

Blood samples were collected in lithium heparinate-containing vials in order to prepare serum samples. Serum testosterone levels were evaluated at day 7 in each group using a RIA kit (Orion Diagnostica, Finland). In addition, circulating levels of GLPG0492 were quantified at steady-state at 3 or 5 h post-dose by LC-MS/MS in Galapagos bio-analytical department.

### RNA analysis

Total RNA was extracted from tibialis using Qiagen RNA extraction kits following manufacturer’s instructions. Total RNA was treated with DNase I (Ambion Inc., Austin, Texas, USA) at 37°C for 30 minutes, followed by inactivation at 75°C for 5 minutes. Real time quantitative PCR (RT-QPCR) assays were performed using an Applied Biosystems ViiA7 sequence detector. Total RNA (1 μg) was reverse transcribed with random hexamers using Taqman reverse-transcription reagents kit (Applied Biosystems) following the manufacturer’s protocol. Gene expression of Atrogin-1, MurF1, IGF1, LC3, FoxO1, IL1β, Myogenin, and PGC1α and PGC1α4 levels were determined by Sybr green assays[29]. Atrogin-1, MurF1, IGF1, LC3, FoxO1, IL1β, Myogenin, PGC1α, PGC1α4 isoform were detected using QuantiTect Primer Assays (Qiagen) while PGC1α4 isoform was detected using forward and reverse primers describe by Ruas and coworkers[30]. GAPDH transcript was used as an internal control to normalize the variations for RNA amounts. Gene expression levels are expressed relative to GAPDH mRNA levels. All the results presented are expressed as mean ± S.E.M. All the primers used in this study and Ct values are available upon request.

### Tissue weighing and fiber cross-sectional area (FCSA) determination

At sacrifice, tibialis and prostate were collected and weighed. Muscles were subsequently snap-frozen in liquid nitrogen. Cryo-sections (10 μm thick) were prepared from the mid-gastrocnemius using a cryostat (Microm) and fixed in formalin for one minute prior assayed with a double immunochemistry IHC as following: sections were probed simultaneously for 30 minutes with a mouse anti-Myosin light chain (Clone NOQ, Abcam) and a rabbit anti-laminin antibody using an autostainer (Dako). Secondary antibodies were anti-mouse-fluorescein and anti-rabbit-dye light, respectively. Laminin IHC provided a fluorescent staining of the border of all fibers and myosin light-chain IHC gave a fluorescent stain of slow-twitch fibers. Consequently, the fibers only immunostained for laminin and not for myosin light chain correspond to the fast-twitch fibers. Histomorphometric analyses were performed using a fluorescent microscope (Objective 20, Olympus). Two series of images were recorded from two representative areas containing either fast-twitch only or both slow and fast-twitch fibers. Morphometric analyses were performed using the imaging software system (Sisn’Com, France). For the evaluation of FCSA and fiber-type distribution, all fibers in each muscle section were analyzed. For FCSA, fibers were gathered in different groups according to their range size, and each group was expressed as a percentage of the total fiber number.

### ^1^H-NMR metabolic profiling

Serum samples (obtained at necropsy 5 h post last dose) were mixed with an equal volume of 3/2 chloroform-D/Methanol-D4. Following incubation, each sample was centrifuged at 6,000 rpm for 10 minutes. The upper layer was recovered and mixed with an internal standard solution provided by Chenomx (Edmonton, Canada). NMR spectra were acquired on a Varian INOVA 800 MHz NMR spectrometer equipped with a Z-gradient HCN 5 mm cold-probe. The pulse sequence used was 1D-noesy with a 990 ms pre-saturation on water and a 4 s acquisition time. Spectra were collected with 416 transients and 4 steady-state scans at 298 K. Spectra were processed and analyzed using Chenomx NMR suite 7.1.

### Statistical analysis

Results are shown as mean ± S.E.M. Data were analyzed by ANOVA followed by Dunnett’s multiple comparison tests. Differences with p < 0.05 were considered to be statistically significant. For FCSA distribution, the relationship between treatment and FCSA distribution has been displayed in a contingency table. The differences between the frequencies of FCSA distribution were tested by Chi-square test. In addition, ANOVA followed by Dunnett’s multiple comparison tests have been performed for each cluster of distribution.

## Results & discussion

**Figure 1:**
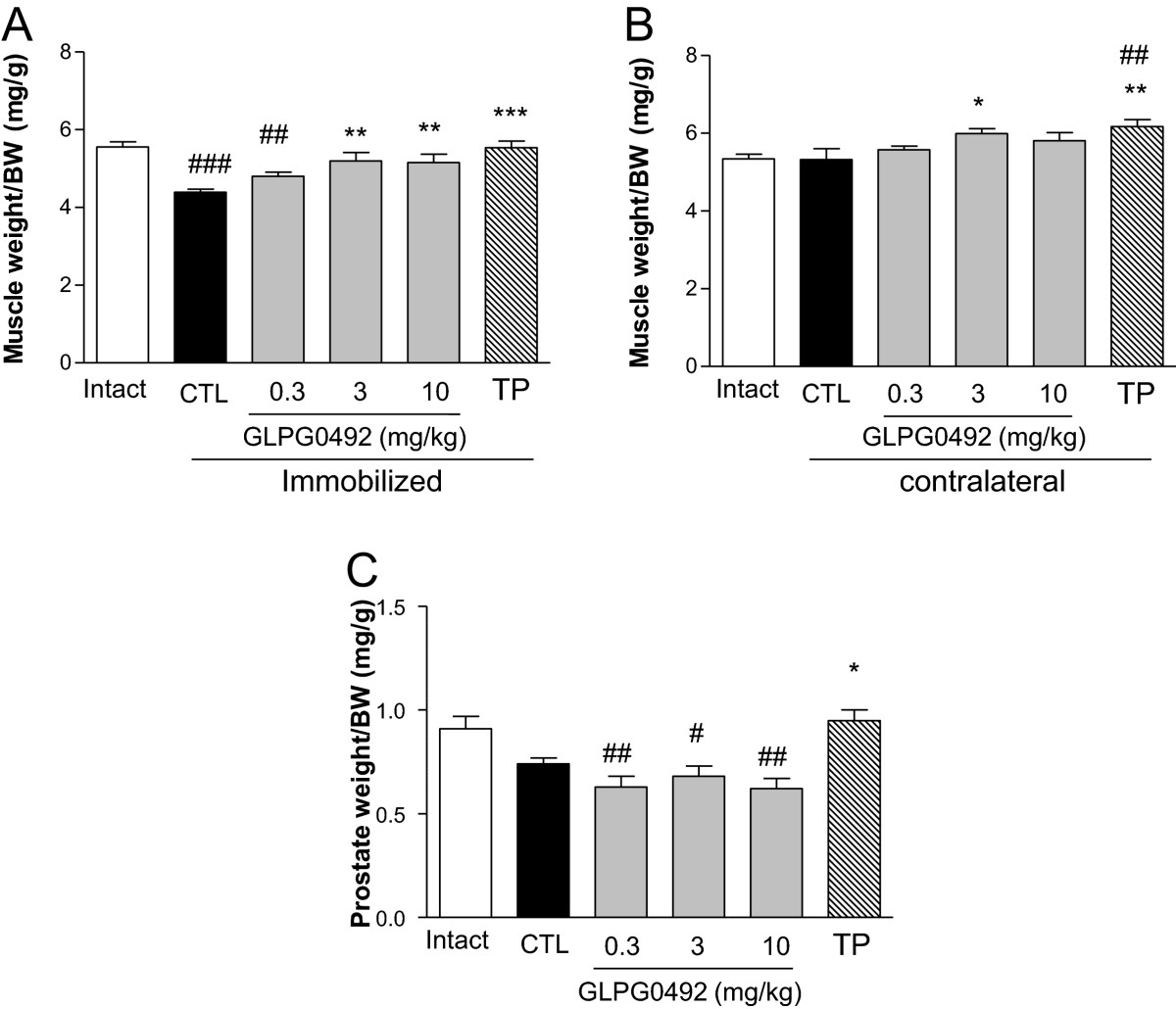
**GLPG0492 reversed immobilization-induced muscle atrophy in a dose-dependent manner.** Normalized gastrocnemius weight from both immobilized (Panel **A**) and contralateral (Panel **B**) legs was assessed at day 7 in intact or immobilized mice receiving increasing doses of GLPG0492 (0.3, 3, 10 mg/kg/day) or TP (1 mg/kg/day) or CTL immobilized (ethanol/corn oil, 5/95 v/v). Panel **C**: Normalized prostate weight at day 7, from immobilized mice receiving increasing doses of GLPG0492 (0.3, 3, 10 mg/kg/day) or TP or CTL vehicle (n = 10 per group; #p < 0.05, ##p < 0.01, ###p < 0.001 vs. Intact (white bar); *p < 0.05, **p < 0.01, ***p < 0.001 vs. CTL immobilized (black bar)).

Skeletal muscle atrophy can be experimentally induced in rodents using different approaches such as the denervation[31], tail suspension[32] or cast immobilization[33]. The anabolic properties of the recently identified SARM, GLPG0492, were evaluated in the adapted mouse model of hindlimb immobilization as described by Okamato and coworkers[28]. In this model, mice are subjected to unilateral hindlimb plaster casting. As expected, hindlimb immobilization for 7 days resulted in a significant loss in muscle mass (-21%, p < 0.001) compared to intact animals (Figure 1A). TP treatment completely prevented muscle loss at day 7 in line with previous reports[34, 35]. Interestingly, GLPG0492 dose-dependently reduced immobilization-induced gastrocnemius atrophy with a maximal significant effect observed between 3 and 10 mg/kg/d. After 7 days of treatment, TP induced a small but significant increase in gastrocnemius weight in the contralateral leg as compared to intact animals, demonstrating its anabolic properties (Figure 1B). A similar dose–response was observed in GLPG0492-treated mice although the difference was found statistically significant only at dose 3 mg/kg/d (Figure 1B). Similar results were obtained in female mice (data not shown). Dose-dependent effects of GLPG0492 in this model are in line with its plasma exposure at steady state (Table 1). These results indicate that GLPG0492, similarly to TP, is able to reduce skeletal muscle atrophy in the hindlimb immobilization model in a dose-dependent manner. It is noteworthy that GLPG0492 efficacy was achieved without significant alterations in circulating testosterone levels (Table 2), nor increasing of prostate weight in contrast to TP as compared to immobilized control group (Figure 1C). Finally if body weight loss is observed between intact group and the whole of immobilized groups at day 7, nor TP, neither GLPG0492 treatments are able to counteract this loss (Table 2).

**Table 1:** **Plasma GLPG0492 levels in male mice at steady-state (n = 5 per group)**

Dose (mg/kg/d)	Time point (hr)	GLPG0492 mean ± SEM (ng/mL)
0.3	3	8.62 ± 2.33
	5	4.78 ± 0.46
3	3	78.8 ± 9.06
	5	58.0 ± 7.45
10	3	184.0 ± 15.4
	5	512.0 ± 324.5

**Table 2:** **Serum testosterone levels and body weight evolution in male mice treated for 7 days with increasing doses of GLPG0492 and TP**

	Testosterone (nM)	Body weight day 0 (gr)	Body weight day 7 (gr)	% BW increase (d7-d0)
Intact	2.4 ± 0.6	24.3 ± 0.3	25.5 ± 0.3	+5.0%
CTL	1.7 ± 0.3	24.2 ± 0.4	22.5 ± 0.5^###^	-6.8%
GLPG0492 0.3 mg/kg/d	1.9 ± 0.6	24.2 ± 0.2	23.7 ± 0.3^###^	-1.9%
GLPG0492 3 mg/kg/d	2.2 ± 0.8	23.6 ± 0.4	22.9 ± 0.3^###^	-2.3%
GLPG0492 10 mg/kg/d	1.7 ± 0.6	23.8 ± 0.2	23.3 ± 0.3^###^	-2.1%
TP 1 mg/kg/d	3.3 ± 0.5	23.8 ± 0.3	23.4 ± 0.4^###^	-1.6%

Data (n = 10 per group) are expressed as mean ± SEM. ^###^p < 0.001 vs. Intact (day7).

**Figure 2:**
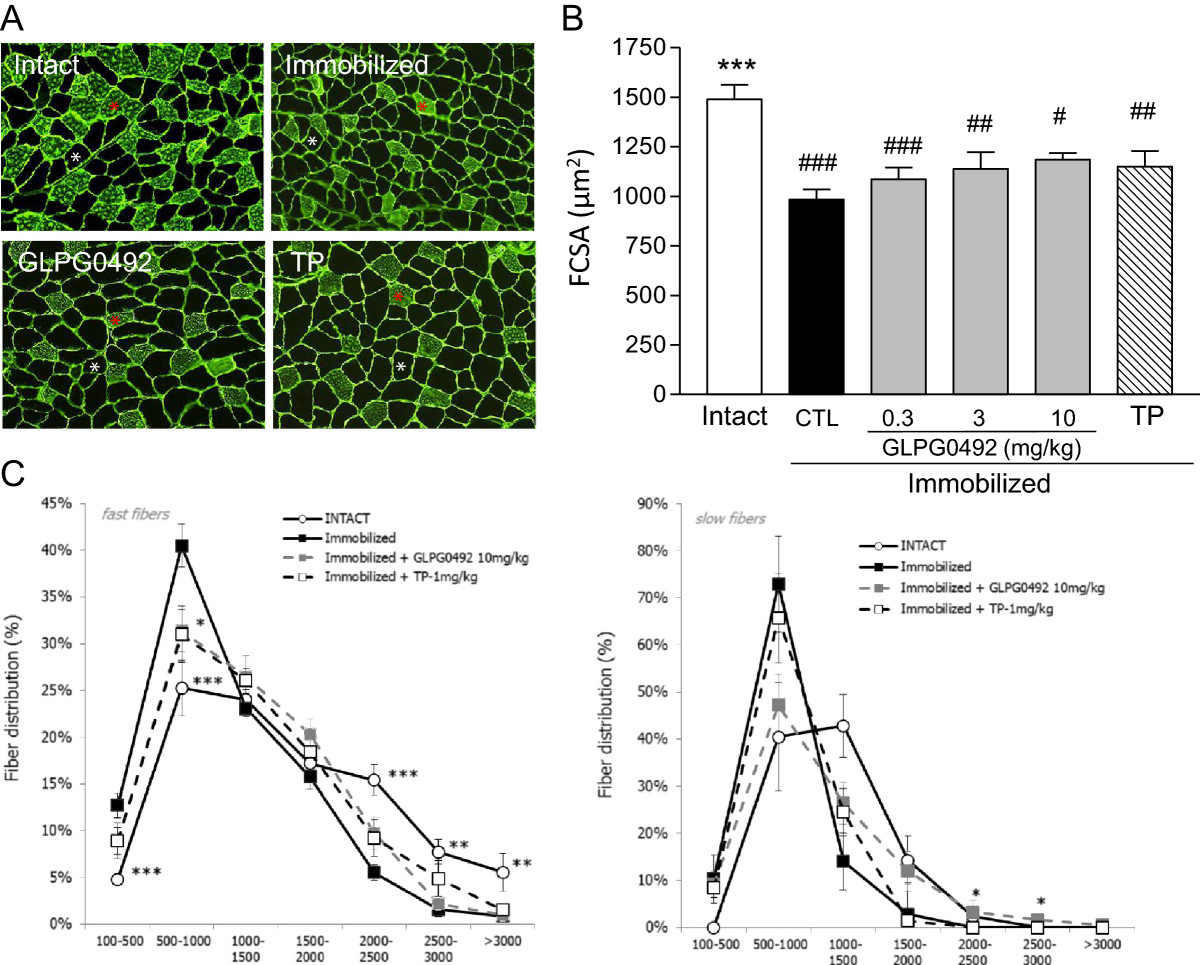
**GLPG0492 reduced muscle fibers atrophy induced by hindlimb immobilization.** Panel **A**: representative immuno-fluorescence staining of gastrocnemius cryosections for myosin light chain and laminin discriminating slow-twitch fibers (red star) from fast-twitch fibers (white star). TP and GLPG0492 were tested at 1 and 10 mg/kg/day, respectively. Panel **B**: FCSA was determined at day 7 in intact and immobilized mice receiving increasing doses of GLPG0492 (0.3, 3, 10 mg/kg/day) or TP (1 mg/kg/day) or CTL vehicle. (n = 10 per group; #p < 0.05, ##p < 0.01, ###p < 0.001 vs. intact; ***p < 0.001 vs. CTL immobilized). Panel **C**: Cross-sectional area (CSA) of gastrocnemius muscle fast and slow fibers distribution (n = 10 per group; *p < 0.05, **p < 0.01, ***p < 0.001 vs. CTL immobilized).

Having established the anabolic properties of GLPG0492 in an experimental model of muscle loss, we next determined its influence on muscle fibers atrophy. As expected, 7 days of immobilization led to a reduction of both slow (indicated by a red star) and fast-twitch (indicated by a white star) fibers diameter as demonstrated by histomorphometric analysis (Figure 2A). These results are in line with previously published reports[28, 36, 37]. Fibers atrophy was similarly modulated by both GLPG0492 (at 10 m/kg/d) and TP treatments (Figure 2B). Effects obtained with GLPG0492 tended to be dose-dependent (Figure 2B). GLPG0492 and TP treatments both modify mean FCSA and distribution of muscle fibers. Moreover FCSA from TP and GLPG0492-treated groups were not significantly different, even though GLPG0492 seems more active on slow fibers (Figure 2B &2C).

These results indicate that GLPG0492 partially prevents immobilization-induced muscle loss by, at least in part, limiting muscle fiber atrophy. It is noteworthy that both GLPG0492 and TP affect similarly both slow and fast twitch muscle fiber in line with previous studies performed in young men[38].

**Figure 3:**
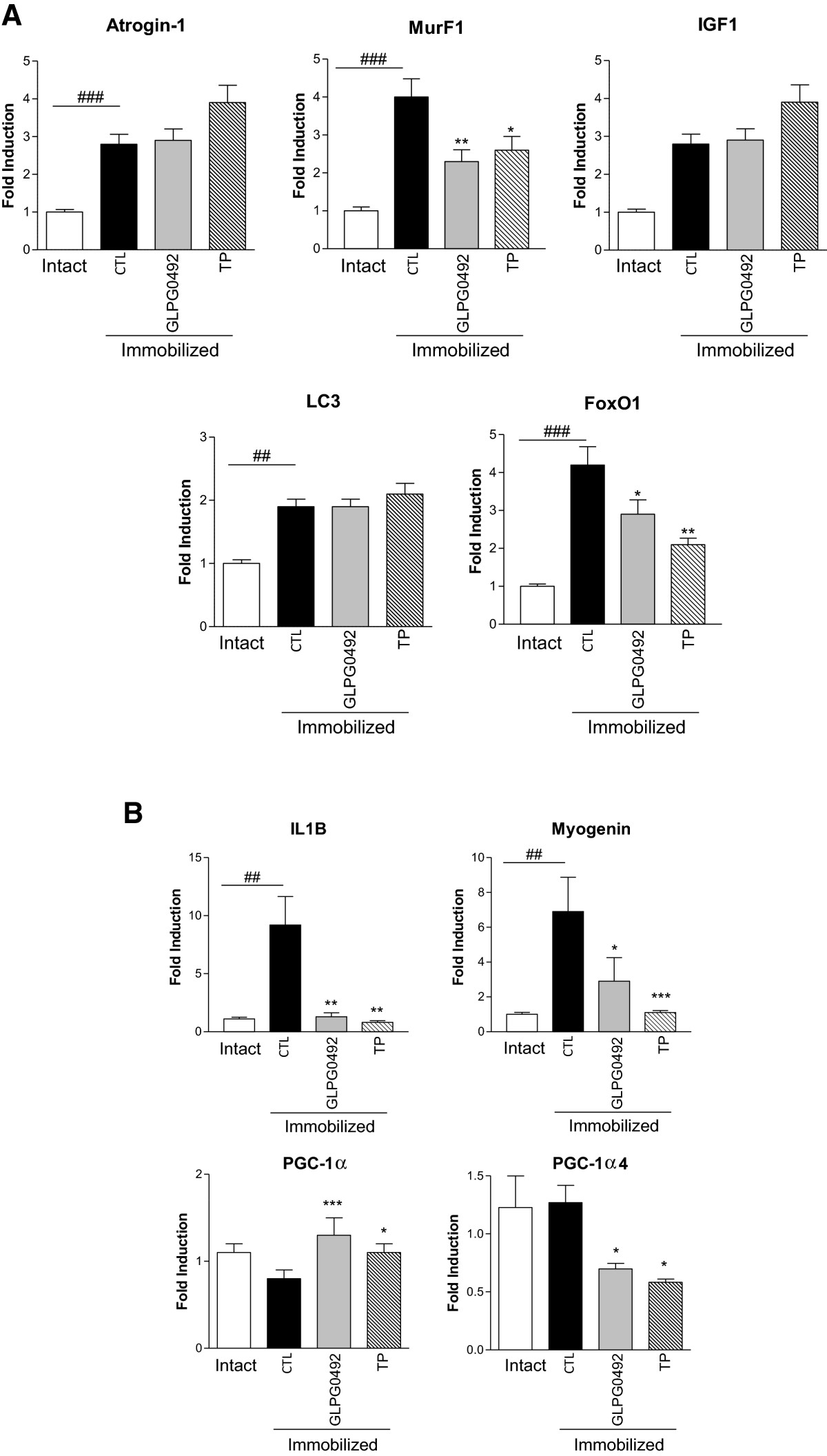
**GLPG0492 selectively modulated key pathways involved in muscle atrophy.** Gene expression levels were monitored at day 7 in tibialis muscle samples from intact or immobilized mice receiving either GLPG0492 (10 mg/kg/day) or TP (1 mg/kg/day) or vehicle. Figure 3a: Atrogin-1, MurF1, LC3, IGFI, FoxO1 and Figure 3b: Myogenin, IL1B, PGC-1α (n = 10 per group; ##p < 0.01, ###p < 0.001 vs. Intact; *p < 0.05, **p < 0.01, ***p < 0.001 GLPG0492-treated vs. CTL immobilized).

Since skeletal muscle atrophy has clearly been linked to the activation of various transcriptional programs (See[4] for review), we next determined the influence of both GLPG0492 and TP on the expression of key genes previously identified in experimentally-induced muscle atrophy[5, 8, 36, 39]. Most of the transcriptional changes linked to atrophy were found to mainly occur early on in the early phases (between day 2 and day 7) in both rodent and human studies[5, 17, 28, 36, 40]. Gene expression studies were performed on tibialis, one of the two intermediary twitch muscles collected. As expected, casting resulted in a significant induction of the expression of the muscle-specific ubiquitin ligases, Atrogin-1 and MurF1 (Figure 3a). Surprisingly, MurF1, but not Atrogin-1, expression was significantly inhibited in response to both GLPG0492 and TP treatments (Figure 3a). Expression of FoxO1, a transcription factor, controlling atrogene expression and maintaining the atrophied state[8, 15], was also found up-regulated after 7 days of immobilization. This gene induction was significantly attenuated in response to both GLPG0492 and TP treatments. Similarly Myogenin, a gene encoding a transcription factor known to be increased in atrophied muscles[5, 41], was also up-regulated after 7 days of immobilization and was also repressed by both GLPG0492 and TP treatments (Figure 3b). Cytokines including TNFα and IL1β have been shown to be involved in the pathogenesis of muscle wasting[42]. Here, we found a very significant induction of IL1β expression (9-fold) in immobilized versus intact animals in line with previous findings[39]. Remarkably, this induction was abolished by GLPG0492 and TP treatments. Finally, PGC-1α mRNA levels were found to be modestly reduced in response to 7 days of immobilization (Figure 3B). PGC-1α is a transcriptional co-activator that controls the expression of genes involved in oxidative metabolism and causes many of the changes associated with endurance training, including mitochondrial biogenesis, fiber-type switching, stimulation of fatty acid oxidation, angiogenesis and resistance to muscle atrophy[43, 19]. So, these results are somehow in contrast with previous reports describing a profound suppression of PGC-1α expression[5, 19, 39]. This discrepancy might be explained by the different kinetic of analysis. Indeed, PGC-1α gene suppression seems to reach its peak after 2 to 3 days of immobilization[5]. In addition, it has been recently described that PGC-1α plays a role in atrophy through the alternative splice variant called alpha-4[30]. Our results shown that expression of PCG-1α4 variant was not regulated by immobilization at day 7. It is noteworthy that gene regulations of the two PGC-1α splice variants, in our model, are small in term of fold induction Nevertheless, the in PGC-1α expression was significantly increased whereas PGC1α4 is significantly decreased by GLPG0492 and TP treatments (Figure 3b). Taken together, these results suggest that GLPG0492 prevents muscle atrophy in this model by, at least in part, negatively interfering with different transcriptional programs related to muscle wasting.

Since skeletal muscle plays a key role in metabolic and energy homeostasis and since limb immobilization has been shown to profoundly affect bioenergetic pathways linked to mitochondrial function[17], we next performed a focused metabolomic analysis using ^1^H-NMR to capture these metabolic changes in the blood compartment and to determine whether GLPG0492 treatment may to some extent affect these alterations of the metabolome. Furthermore, identified metabolites significantly modulated in blood in response to GLPG0492 treatment might be considered as potential biomarkers of GLPG0492 during clinical development. Using ^1^H-NMR, 40 metabolites could be identified and precisely quantified within these samples (Table 3). The BCAAs are among the nine essential amino acids for humans, accounting for 35% of the essential amino acids in muscle proteins and 40% of the preformed amino acids required by mammals. Although it has been reported that muscle protein synthesis significantly declined during the first 6 h of immobilization (40), it has also been shown that immobilization of hindlimb by cast fixation for 1 d increased muscle protein breakdown[44]. Here, our hindlimb immobilization model led to a notable, but non-significant reduction in a number of circulating amino acids including the so-called branched-chain amino acids (BCAA: isoleucine, leucine and valine). BCAA have been shown to inhibit proteolysis in skeletal muscle[45, 46]. These amino acids provide the amino groups required for synthesis of glutamine and alanine. Protein degradation in skeletal muscle results in the release of amino acids, particularly alanine. But alanine is channeled to the liver for gluconeogenesis and APP synthesis, and ALT activity is increased during immobilization, which could explain the reduced plasma alanine concentration in immobilized animals[47]. In addition, urocanate, a product derived from histidine breakdown, was significantly increased in serum samples from immobilized mice (Table 3).

**Table 3:** **List of metabolites identified by** ^**1**^ **H-NMR analysis of serum samples from intact or immobilized mice after 7 days of treatment with GLPG0492 (10 mg/kg/d) or CTL**

	Intact		Immobilized					
	Mean	SEM	CTL			GLPG0492		
			Mean	SEM	p-value	Mean	SEM	p-value
3-Hydroxybutyrate	239	46	184	25		1207	302	**
Acetate	542	92	460	6		857	124	*
Acetone	19	5	14	3		54		*
Alanine	1977	156	1214	191	##	1218	45	
Asparagine	201	27	124	21	#	127	10	
Betaine	142	32	79	32		114	14	
Choline	222	24	189	24		251	34	
Citrate	773	116	597	63		1076	169	*
Creatine	817	40	839	132		891	97	
Dimethylamine	13	1	11	2		10	1	
Ethanol	220	31	146	34		181	13	
Formate	174	23	155	10		165	27	
Fumarate	20	3	12	4		33	7	*
Glucose	29159	4531	23873	3222		33088	2902	
Glutamate	337	37	256	37		334	27	
Glutamine	1595	239	1019	125		1701	147	*
Glycerol	2638	238	2366	377		3010	469	
Glycine	1400	138	893	130	#	1195	102	
Histidine	248	27	169	32		218	17	
Isobutyrate	57	10	30	4	#	49	6	
Isoleucine	393	59	244	37		286	26	
Lactate	19808	1196	13878	1491	#	16821	1797	
Leucine	486	76	337	52		369	39	
Lysine	744	49	792	100		737	81	
Malonate	89	13	80	9		98	10	
Mannose	106	15	136	28		251	35	*
Methionine	341	34	212	34	#	207	18	
O-Acetylcarnitine	45	10	39	5		105	17	**
Phenylalanine	235	20	179	27		176	11	
Proline	627	47	337	66	##	270	41	
Pyruvate	480	23	261	32	#	471	65	*
Serine	740	67	449	64	##	524	29	
Succinate	44	9	44	5		85	20	
Taurine	2811	217	2860	452		3498	299	
Threonine	938	85	636	117		631	34	
Trimethylamine N-oxide	200	29	143	41		134	24	
Tryptophan	83	7	65	8		52	11	
Tyrosine	252	19	171	23	#	168	8	
Urocanate	0	0	32	11	#	44	5	
Valine	955	136	598	93		610		

#p < 0.05, ##p < 0.01 immobilized vs. intact; *p < 0.05, **p < 0.01 GLPG0492-treated vs. CTL; Data are expressed as mean concentrations (μM) ± SEM.

**Figure 4:**
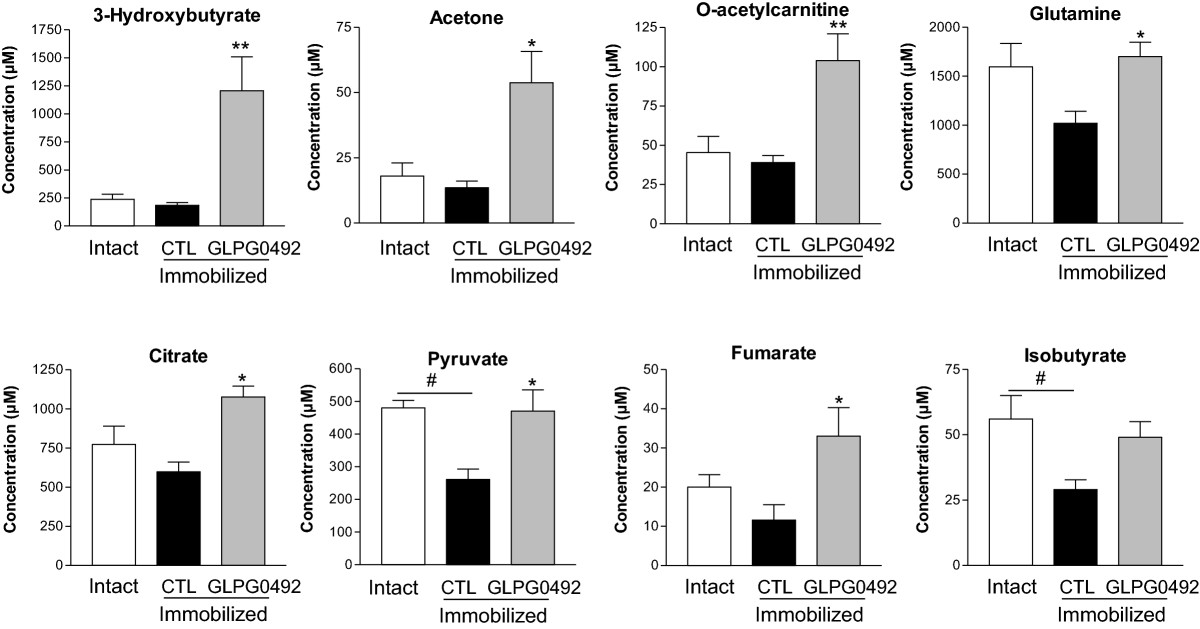
**GLPG0492 increased plasma metabolite levels linked to TCA cycle, β - oxidation and amino acids metabolism.** Metabolic profiling by 1H-NMR of serum samples from intact or immobilized mice receiving either GLPG0492 (10 mg/kg/day) or vehicle was performed at day 7. (n = 5 per group; #p < 0.05 Intact vs. Immobilized; *p < 0.05, **p < 0.01 vs. CTL immobilized).

As in our study, increased of atrogin-1 and MuRF1 proteins have been reported in hindlimb suspension rat model. L-leucine and the leucine metabolite β-hydroxy-β-methylbutyrate (Ca-HMB) have been reported to attenuate the increase in atrogin-1 and MuRF1 proteins in hindlimb suspension rat model[48]. Another recent paper suggest that leucine, could down regulate the MurF1 protein in mouse cachexia model[49]. Nevertheless, in our study, modulation of BCAAs, especially leucine, by GLPG0492 is not strong enough to explain the significant gene regulation of MurF1. *In fine*, GLPG0492 treatment for 7 days did not significantly alter these changes in amino-acids levels with one exception being glutamine (Figure 4; Table 3). Glutamine has been shown to stimulate protein synthesis and to inhibit protein catabolism in skeletal muscle *in vitro* and *in vivo*[47].

By contrast, GLPG0492 treatment led to a significant elevation in tricarboxylic acid (TCA) cycle intermediates such as pyruvate, citrate, fumarate and succinate to a lesser extent (non-significant, p = 0.054) (Table 3; Figure 4). These TCA cycle intermediates were found as part of a metabolic signature of exercise in human plasma[50] and clearly point to a modulation of cellular bioenergetic processes linked to mitochondrial function. Moreover, ketone bodies such as 3-hydroxybutyrate, acetate and acetone were found also to be increased in response to our SARM. Interestingly, Vega and coworkers reported a similar increase in 3-hydroxybutyrate in adult men in response to a short-term administration of oxandrolone, a synthetic testosterone derivative[51]. These authors suggested that oxandolone induced hepatic ketogenesis. Additional studies will be required to determine whether GLPG0492-mediated 3-hydroxybutyrate elevation in this hindlimb immobilization model is occurring through a similar mechanism. Finally, O-acetylcarnitine levels were doubled in response to GLPG0492. This might reflect an increase in fatty acid oxidation. Interestingly, plasma acetylcarnitines were identified as exercise-related biomarkers and to be essentially derived from skeletal muscle[52]. Taken together, results from the focused metabolomic study led to the identification of GLPG0492-specific metabolomic signature in the hindlimb immobilization model.

Our results are in accordance with several studies in rodents suggesting that muscle atrophy results from a large increase in proteolysis and an increased oxidative stress. Unfortunately, little information is available about the influence of immobilization on markers of muscle protein breakdown and oxidative stress in humans.

Results of these investigations indicate that GLPG0492, a non-steroidal SARM, is able to significantly reduce muscle atrophy in a mouse hindlimb immobilization model. These protective effects were dose-dependent, and achieved without significant alteration in serum testosterone levels, neither prostate weight (Figure 1C and Table 2). Moreover, the magnitude of the observed effects on muscle was comparable to the impact of TP. Muscle anabolic activities of SARM and testosterone are well-established in man[20, 21, 53, 54] and confirm in our model with significant increase of normalized prostate weight by TP. The molecular mechanisms by which AR ligands promote anabolism in skeletal muscle are very diverse and are still a matter of debate (See[54] for review). Androgens are believed to affect muscle mass by regulating protein synthesis, reducing protein breakdown, modulating myogenic differentiation and by interfering with key signaling pathways involved in skeletal muscle homeostasis[54]. These effects are mainly mediated by the AR. Chambon and colleagues demonstrated that the anabolic effects of androgens are transduced in limb muscles by the AR expressed in myocytes but also by satellite cells and myofibroblasts[55, 56]. Moreover, this group reported a significant impact of AR gene deletion in myocytes on FCSA in both fast and intermediary-twitch muscles via a regulation of IGF-IEa production. In addition, myofibril organization was found to be disrupted in muscle-specific AR-deficient mice[55, 56]. On the other hand, McLean and coworkers reported the characterization of AR total knock-out mice[57]. Male ARKO mice displayed a significantly reduced skeletal mass which could not be linked to a modulation of IGF-1 signaling[57]. In this report, transcriptomic analysis suggested that androgens regulate muscle mass via the control of muscle commitment and proliferation[57]. These effects might be explained, at least in part, by the AR-dependent regulation of ornithine decarboxylase, which regulates both myoblast proliferation and delays differentiation as demonstrated by a reduced myogenin expression[58]. In this model of hindlimb immobilization, we showed that both TP and GLPG0492 were able to prevent muscle atrophy by down-regulating key transcriptional programs induced by muscle disuse (Figure 3)[4]. Gene expression studies revealed the complex interplay between AR-dependent signaling pathways (See[50] for review). Interestingly, we did not observe any regulation of IGF-1Ea gene expression (Figure 3A). By contrast, myogenin expression which was significantly increased following immobilization in line with previous studies[5, 39], was completely suppressed in response to both GLPG0492 and TP treatments suggesting a potential impact on myoblast differentiation[58]. As previously described by Caron and coworkers in the immobilized mouse model, the induction of the muscle-specific ubiquitin ligases, MurF1 and Atrogin-1 is a hallmark of the atrophied state[6, 7, 36]. Surprisingly, both GLPG0492 and TP were found to inhibit immobilization-induced MurF1 but not Atrogin-1 expression (Figure 3). This selective regulation of these ubiquitin ligases is reminiscent of the phenotype of the muscle-specific IKKβ transgenic mice[59]. Over-expression of IKKβ in the muscle leads to severe muscle wasting due to MurF1 but not Atrogin-1 gene induction[59]. The role of NFκB signaling in muscle atrophy was independently confirmed by Hunter and colleagues[60, 61]. Since AR has been shown to negatively interfering with NFκB signaling pathway[60], it is tempting to speculate that both GLPG0492 and TP inhibit MurF1 expression via this mechanism. Further studies are required to fully establish this cross-talk in the atrophied muscle. Nevertheless, the inhibition of MurF1 expression suggests that GLPG0492 and TP prevent muscle atrophy, by slowing-down protein catabolism.

Interestingly, both treatments failed to regulate markers of autophagy as demonstrated by the lack of LC3 gene regulation despite a significant induction following immobilization for 7 days (Figure 3A). While the role of TNFα and other cytokines in disuse-induced atrophy remain unclear[62], we found a strong up-regulation of IL1β mRNA levels following immobilization in line with previous results[39]. This induction was completely abolished by treatment with both TP and GLPG0492 (Figure 3) suggesting again a potential cross-talk between NFκB and AR pathways at the transcriptional level. FoxOs have been identified as the major transcription factors controlling the balance between protein catabolism and anabolism[4]. FoxO1 expression is significantly up-regulated in our model of hindlimb immobilization (Figure 3A) in line with previous reports[5, 8, 15]. This induction is also significantly repressed by both GLPG0492 and TP treatments suggesting that AR activation may prevent muscle-atrophy by negatively interfering with this transcriptional pathway leading ultimately to a regulation of protein homeostasis in this tissue. It is noteworthy that FoxO signaling has been shown to be repressed by PGC-1α[19] whose expression was found to be increased in response to GLPG0492 and TP to a lesser extent (Figure 3A). Taken together, these results indicate that GLPG0492 minimizes muscle atrophy by antagonizing the key transcriptional pathways governing both anabolic and catabolic response.

Results obtained in the focused metabolomic study points to a modulation of cellular bioenergetic processes linked to mitochondrial function and perhaps to ketogenesis (Table 3; Figure 4). These results are quite consistent with the observed up-regulation of PGC-1α gene expression and may reflect the overall cellular state upon chronic treatment with GLPG0492 in this hindlimb immobilization model. In addition, the GLPG0492 metabolomic signature is somehow comparable to signatures previously related to moderate exercise in human plasma[50, 52]. Further studies will be required to fully demonstrate that these alterations of the plasma metabolome are mainly reflecting changes in skeletal muscle physiology. More studies are still required to investigate the possibility that muscle protein synthesis could be assessed as a plasma biomarker in human cachexia. In a recent study, Glover and coworkers have measured markers of protein breakdown and oxidative stress in muscles of subjects who underwent 14 days of knee-brace-mediated immobilization. These static measures of breakdown and oxidative modifications suggested that a small increase in protein ubiquitination occurs early (2 days post-immobilization), but is not sustained during the later phase (after 14 days) of muscle atrophy in humans, suggesting that these pathways are not playing a major role in simple disuse-induced atrophic loss of protein mass[63]. Other groups have hypothesized that the loss of muscle mass results from a reduction in the rate of protein synthesis. Thus, the normal diurnal fasted-to-fed cycle of protein balance is disrupted and, as a consequence proteolysis becomes dominant but is not enhanced[63]. Nevertheless, this metabolomics signature might be considered as a *bona fide* biomarker of target engagement in future clinical studies and may represent an alternative to the measurement of the muscle fractional synthetic rate as a mechanistic endpoint which remains quite challenging to implement in phase 1 studies conducted in young healthy volunteers[64].

## Conclusion

GLPG0492 was found as efficacious as TP at reducing muscle loss in the hindlimb immobilization model. These effects were achieved without any significant androgenic effects on sexual tissue (Table 1, Figure 1C). Collectively, these results unveil the potential of GLPG0492, a non-steroidal SARM, as treatment for musculo-skeletal diseases such as disuse-induced loss of muscle mass (e.g. coma, paralysis, hospitalization, limb immobilization).

## Authors’ original submitted files for images

Below are the links to the authors’ original submitted files for images. Authors’ original file for figure 1 Authors’ original file for figure 2 Authors’ original file for figure 3 Authors’ original file for figure 4 Authors’ original file for figure 5
