# Supplementary figures and images for: Characterization of GLPG0492, a selective androgen receptor modulator, in a mouse model of hindlimb immobilization
Source: BMC Musculoskelet Disord. 2014 Sep 3;15:291. doi: 10.1186/1471-2474-15-291 (PMC4167280; doi:10.1186/1471-2474-15-291)

**A**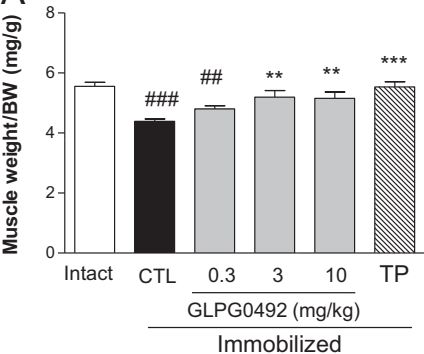**B**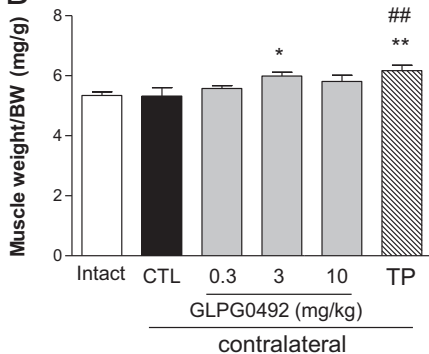**C**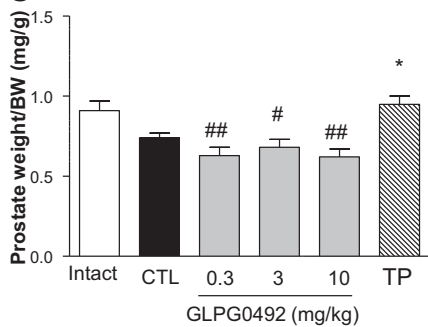

Supplement: Supplementary file 1 — Authors’ original file for figure 1 [file 12891_2014_2241_MOESM1_ESM.pdf]

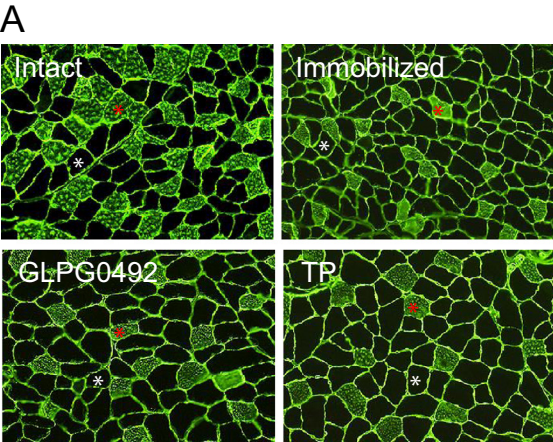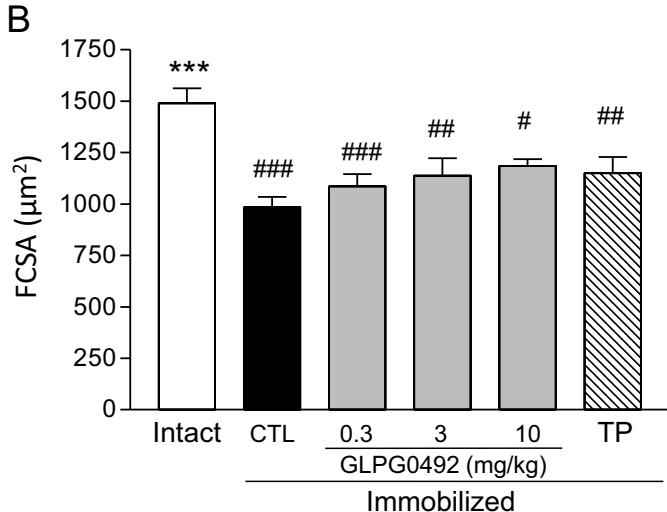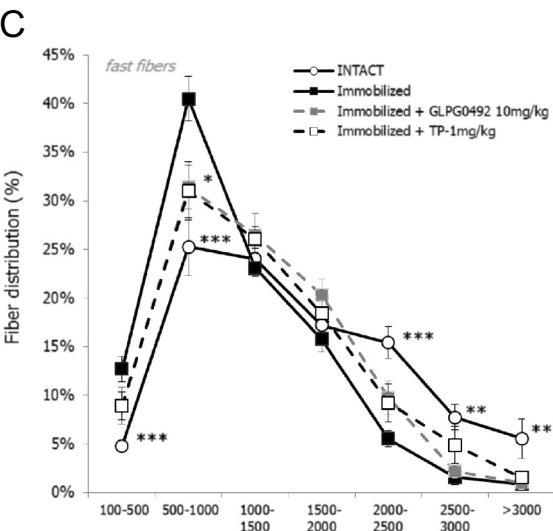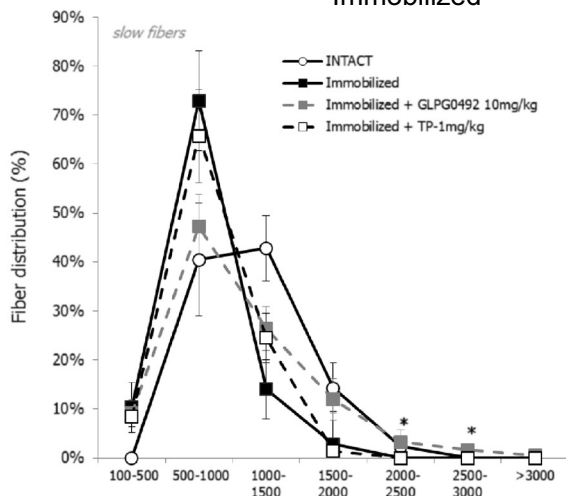

Supplement: Supplementary file 2 — Authors’ original file for figure 2 [file 12891_2014_2241_MOESM2_ESM.pdf]

**A**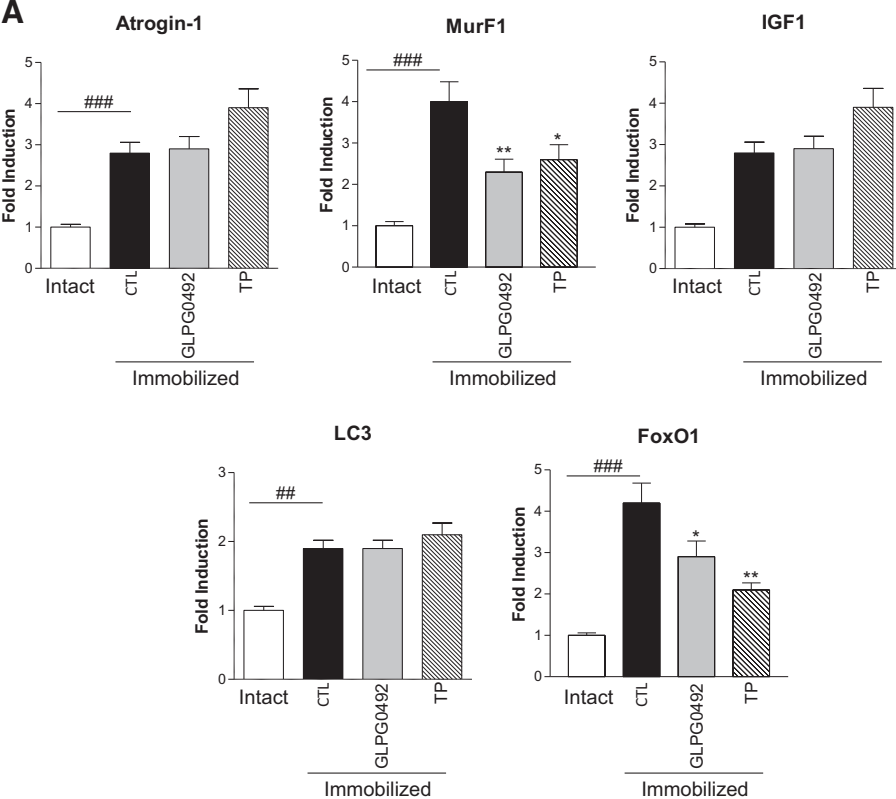**B**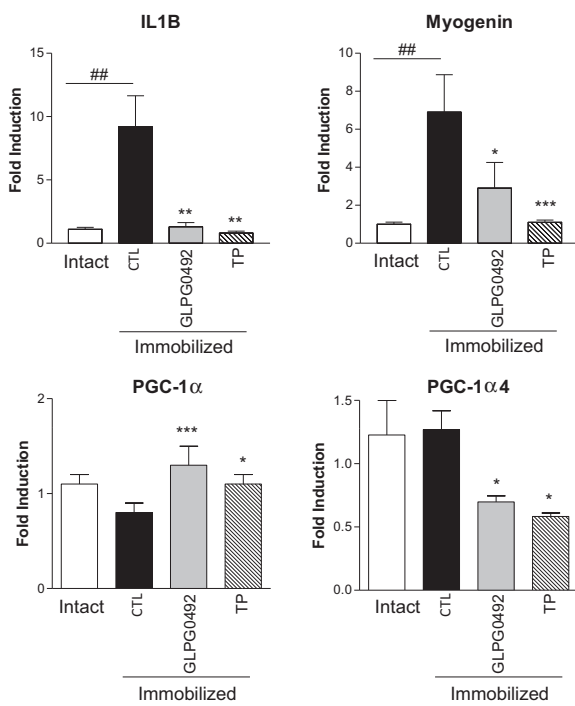

Supplement: Supplementary file 3 — Authors’ original file for figure 3 [file 12891_2014_2241_MOESM3_ESM.pdf]

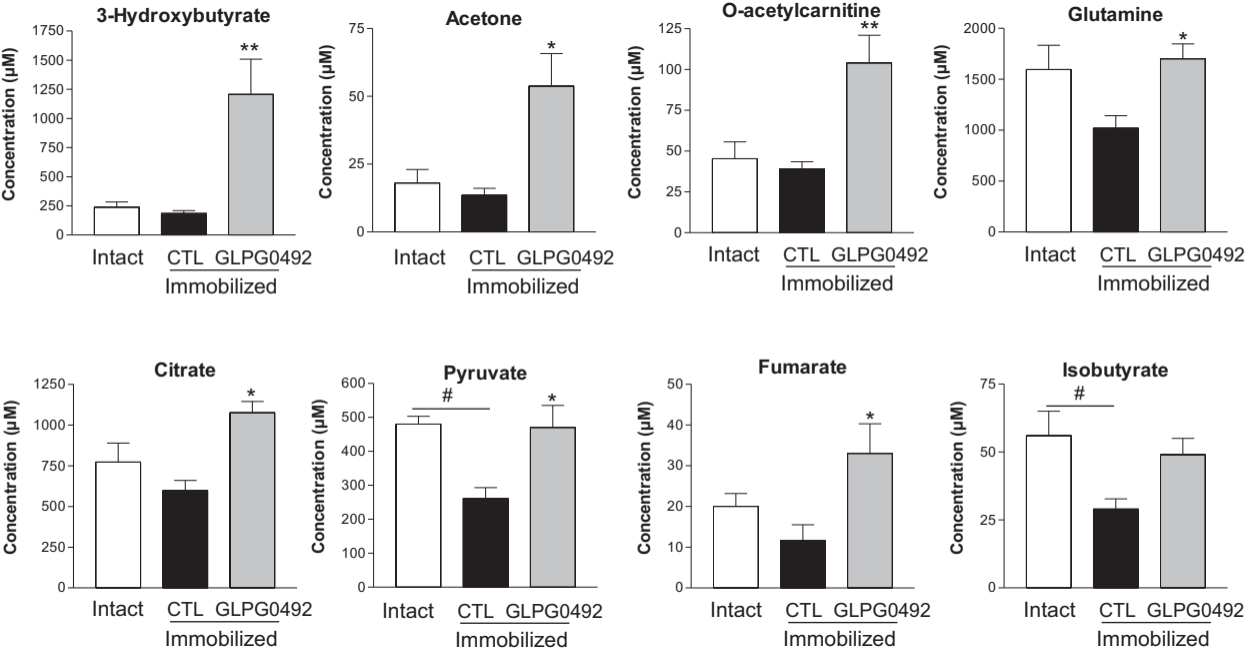

Supplement: Supplementary file 4 — Authors’ original file for figure 4 [file 12891_2014_2241_MOESM4_ESM.pdf]
