# Supplementary material for: Characterization of GLPG0492, a selective androgen receptor modulator, in a mouse model of hindlimb immobilization
Source: BMC Musculoskelet Disord. 2014 Sep 3;15:291. doi: 10.1186/1471-2474-15-291 (PMC4167280; doi:10.1186/1471-2474-15-291)
Supplement: Supplementary file 5 — Authors’ original file for figure 5 [file 12891_2014_2241_MOESM5_ESM.pptx]

## Slide 1
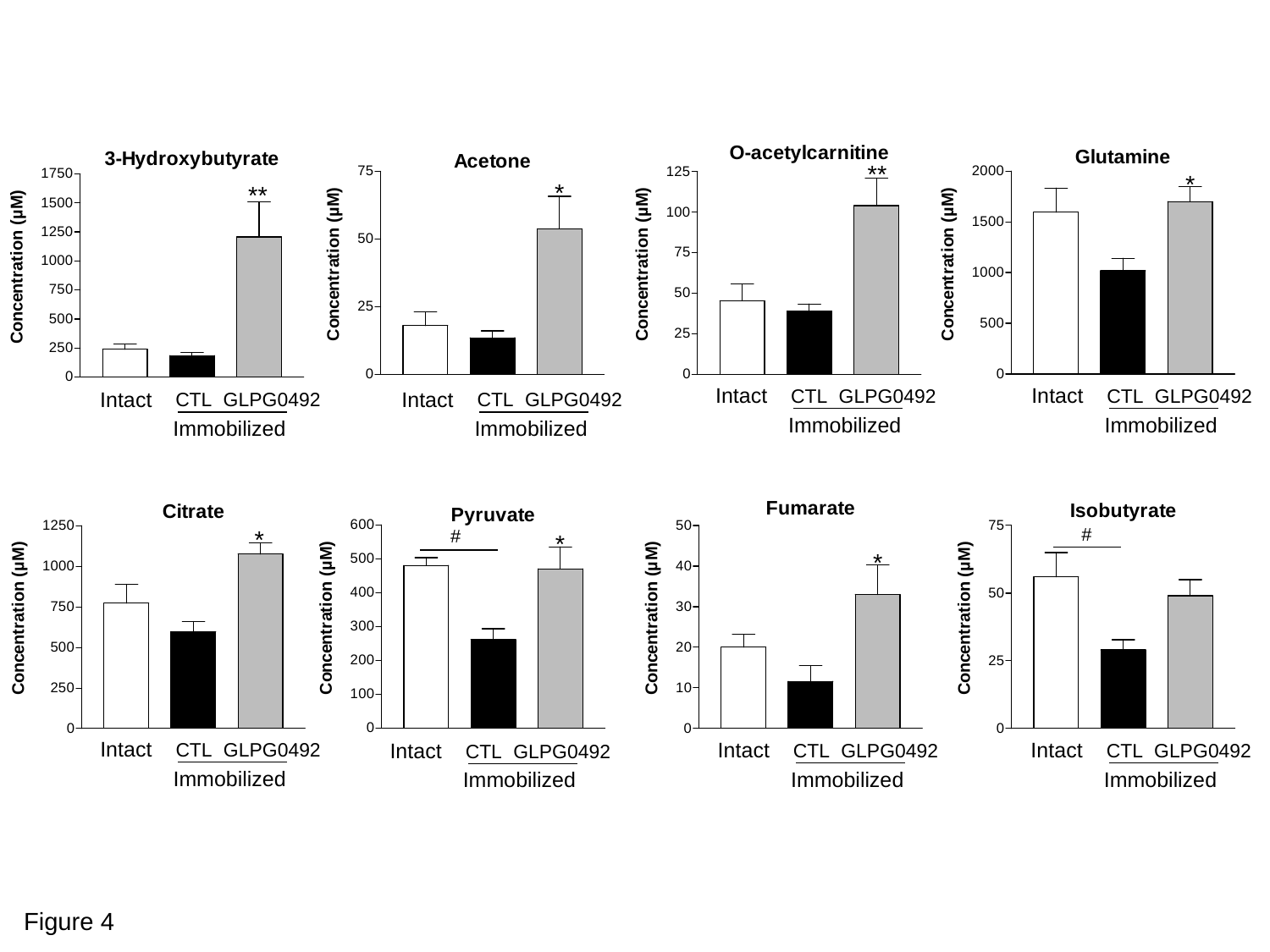

**
*
*
**
Intact
Intact
CTL
GLPG0492
CTL
GLPG0492
Intact
Intact
CTL
GLPG0492
CTL
GLPG0492
Immobilized
Immobilized
Immobilized
Immobilized
#
*
#
*
*
Intact
Intact
Intact
Intact
CTL
GLPG0492
CTL
GLPG0492
CTL
GLPG0492
CTL
GLPG0492
Immobilized
Immobilized
Immobilized
Immobilized
Figure 4
